# The Vacuolating Autotransporter Toxin (Vat) of *Escherichia coli* Causes Cell Cytoskeleton Changes and Produces Non-lysosomal Vacuole Formation in Bladder Epithelial Cells

**DOI:** 10.3389/fcimb.2020.00299

**Published:** 2020-06-26

**Authors:** Juan Manuel Díaz, Charles M. Dozois, Francisco Javier Avelar-González, Eduardo Hernández-Cuellar, Pravil Pokharel, Alfredo Salazar de Santiago, Alma Lilian Guerrero-Barrera

**Affiliations:** ^1^Departamento de Morfología, Universidad Autónoma de Aguascalientes (UAA), Aguascalientes, Mexico; ^2^Institut National de Recherche Scientifique (INRS)-Centre Armand-Fappier Santé Biotechnologie, Laval, QC, Canada; ^3^Departamento de Fisiología y Farmacología, Universidad Autónoma de Aguascalientes (UAA), Aguascalientes, Mexico

**Keywords:** urinary tract infection, *Escherichia coli*, virulence factors, vat, cell damage, vacuoles, cytoskeleton, cell junctions

## Abstract

Urinary tract infections (UTIs) affect more than 150 million people, with a cost of over 3.5 billion dollars, each year. *Escherichia coli* is associated with 70–80% of UTIs. Uropathogenic *E. coli* (UPEC) has virulence factors including adhesins, siderophores, and toxins that damage host cells. Vacuolating autotransporter toxin (Vat) is a member of serine protease autotransporter proteins of *Enterobacteriaceae* (SPATEs) present in some uropathogenic *E. coli* (UPEC) strains. Vat has been identified in 20–36% of UPEC and is present in almost 68% of urosepsis isolates. However, the mechanism of action of Vat on host cells is not well-known. Thus, in this study the effect of Vat in a urothelium model of bladder cells was investigated. Several toxin concentrations were tested for different time periods, resulting in 15–47% of cellular damage as measured by the LDH assay. Vat induced vacuole formation on the urothelium model in a time-dependent manner. Vat treatment showed loss of the intercellular contacts on the bladder cell monolayer, observed by Scanning Electron Microscopy. This was also shown using antibodies against ZO-1 and occludin by immunofluorescence. Additionally, changes in permeability of the epithelial monolayer was demonstrated with a fluorescence-based permeability assay. Cellular damage was also evaluated by the identification of cytoskeletal changes produced by Vat. Thus, after Vat treatment, cells presented F-actin distribution changes and loss of stress fibers in comparison with control cells. Vat also modified tubulin, but it was not found to affect Arp3 distribution. In order to find the nature of the vacuoles generated by Vat, the Lysotracker deep red fluorescent dye for the detection of acidic organelles was used. Cells treated with Vat showed generation of some vacuoles without acidic content. An *ex vivo* experiment with mouse bladder exposed to Vat demonstrated loss of integrity of the urothelium. In conclusion, Vat induced cellular damage, vacuole formation, and urothelial barrier dysregulation of bladder epithelial cells. Further studies are needed to elucidate the role of these vacuoles induced by Vat and their relationship with the pathogenesis of urinary tract infection.

## Introduction

Urinary tract infections (UTIs) are a public health problem that affects more than 150 million people, with an estimated cost of over 3.5 billion dollars each year (Flores-Mireles et al., 2015). For the development of the disease, several risk factors exist such as diabetes, vaginal infections, sexual activity, presence of a urinary catheter, neurological disease, immunosuppression, and kidney transplantation (Foxman, 2003; Nielubowicz and Mobley, 2010).

UTIs are caused mainly by bacteria, but sometimes can be provoked by yeast or other fungi. The most frequent cause of UTIs is uropathogenic *Escherichia coli* (UPEC), with a prevalence of 70 to 80% worldwide (Flores-Mireles et al., 2015; Ramírez-Castillo et al., 2018). *Escherichia coli* is typically found in the gastrointestinal tract as part of the microbiota, and certain commensal *E. coli* strains residing in the gut have the potential to cause UTIs. The difference between purely commensal *E. coli* strains and UPEC is the presence of certain virulence factors in the pathogenic strains (Terlizzi et al., 2017). UPEC has the capacity to attach, colonize and invade the urinary tract through production of several virulence factors including adhesins, siderophores, capsular polysaccharides and the production of toxins (Kaper et al., 2004; Crépin et al., 2012; López-Banda et al., 2014).

One of the virulence factors identified in some UPEC is the Vacuolating autotransporter toxin (Vat), which is a member of the serine protease autotransporter proteins of *Enterobacteriaceae* (SPATEs). The Vat toxin is a ~110 kDa secreted protein exported by the Type Va secretion system and belongs to the class II cytotoxic SPATEs (Henderson and Nataro, 2001; Dutta et al., 2002; Nichols et al., 2016). The *vat* gene has been identified in both avian pathogenic *E. coli* (APEC) and UPEC strains. It has been shown to generate the formation of vacuoles in chicken embryo fibroblasts and contribute to the development of cellulitis in chickens (Parreira and Gyles, 2003). Although the mechanism of action of Vat and its implication in the development of UTIs is not entirely known, the gene sequences encoding the toxin were detected in 36% of UPEC strains (Ramírez-Castillo et al., 2018). In a different study, the *vat* gene was found in patients with cystitis (57.9%), pyelonephritis (59.3%), prostatitis (72.4%) and septicemia (64.7%) (Parham et al., 2005; Spurbeck et al., 2012; Nichols et al., 2016). Also, our group recently published the prevalence of *vat* genes in UPEC from Guadeloupe (Habouria et al., 2019) where *vat* sequences were found in 333 isolates (48.7%) of the UTI strains. Despite the fact that Vat is one of the most prevalent SPATEs in UPEC, the mechanism of action and specific activity of this protein during urinary infection has not been determined (Welch, 2016).

The bladder epithelial cell is an *in vitro* cell culture model extensively studied because of the interaction of these cells during the pathogenesis of infection (McLellan and Hunstad, 2016). The urothelium plays a significant role as a barrier against biotic and abiotic agents, and disruption of this barrier may lead to urinary tract disease (Parsons, 2007). The characteristics of the urothelial wall are mainly imparted by the integrity of the bladder cells. Interestingly, enteroaggregative and enteropathogenic *Escherichia coli* induce epithelial cell damage that involves virulence factors such as SPATEs (Gates and Peifer, 2005; Khurana, 2006; Windoffer et al., 2011; Sanchez-Villamil et al., 2019). Thus, the objective of this study was to determine the effects of the vacuolating autotransporter toxin, Vat, from *Escherichia coli* on human epithelial bladder cells, in order to elucidate the mechanism of action of this toxin, and to serve as a basis for a more detailed study of this virulence factor, serving as a precedent of its function *in vivo*.

## Materials and Methods

### Bacterial Strains and Cell Culture

The *vat* autotransporter encoding gene was amplified by PCR using specific primers (Forward: TATTGGATCCTCCGCTCTGAACCGCCACGC; Reverse: CAAGCTTCGTAATCAGATAATCGCAGC) from pathogenic *E. coli* strain QT598 (Genbank accession QDB64244.1) (Habouria et al., 2019), which with the exception of a single Arg_534_ to His_534_ substitution is identical to Vat (c0393) from UPEC strain CFT073. PCR products contained 15 bp extensions homologous to the pUCmT multi-cloning site. Linearized pUCmT digested with *Xho*I and *BamHI* was used to clone inserts by fusion reaction with the Quick-fusion cloning kit (Biotool, #B2261). The plasmid clones were transformed into *E. coli* DH5α then into *E. coli* BL21 for protein production (Habouria et al., 2019). The model to test the cytotoxicity of Vat was performed with human urinary bladder epithelial cell line ATCC 5637 (American Type Culture Collection HTB-9). The cells were maintained in RPMI 1640 (Sigma-Aldrich, #R7509) supplemented with 10% heat-inactivated fetal calf serum (Invitrogen, #16000044) without antibiotics.

### Vat Production and Concentration

**Figure 1:**
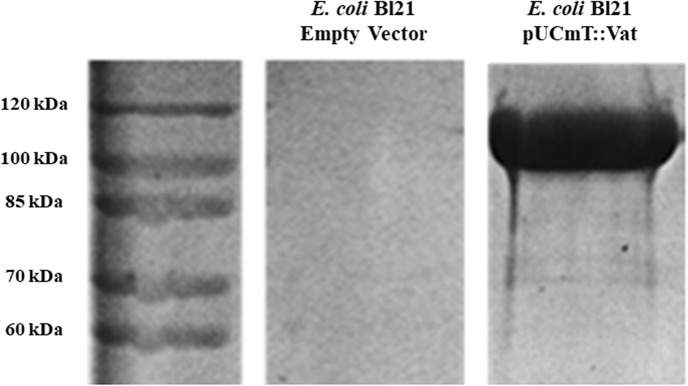
Overexpression of Vat protein detected by SDS-PAGE. The filtered supernatants from overnight cultures of *E. coli* BL21 (pUCmT::vat) and *E. coli* BL21 empty vector were concentrated by Amicon filter units and the protein from the supernatant was migrated next to a protein marker (10–200 kDa). The gel was stained with Coomassie Blue.

*Escherichia coli* BL21 (pUCmT::*vat*) was grown in 200 ml of Lennox Broth (LB) medium with ampicillin (100 μg/ml) overnight (37°C/100 rpm). The culture was centrifuged (2,370 × g for 10 min at 4°C) and the supernatant filtered through a 0.22 μm membrane filter (Corning Inc, # R7509) (Salvadori et al., 2001). The protein from the sterile supernatant was concentrated through a 50 kDa centrifugal filter unit (Millipore, #UFC905024) by centrifugation (2,370 × g for 10 min at 4°C). Quantification of the protein was determined by Bradford assay, and the absorbance was measured at 595 nm, using a microplate reader (Bio-Rad, #028007). To determine the concentration of the protein the measurements were overlapped with a standard linear curve. The protein was visualized using Coomassie blue staining (Figure 1) after separation by sodium dodecyl sulfate-polyacrylamide gel electrophoresis (SDS-PAGE) (Dutta et al., 2002).

### Cytotoxic Effect of Vat Toxin Measured by Lactate Dehydrogenase Release Assay

Confluent cultures of the 5637 cell line were grown in 96-well plates and were treated with different Vat concentrations from the concentrated supernatant sample (5, 25, 50, 75, 100 μg/ml) in a final volume of 100 μl of RPMI per well, and incubated at different times (3, 6, 12, and 24 h) (Dutta et al., 2002). Parreira and Gyles, 2003). The culture supernatant from *E. coli* BL21 containing the empty vector (pUCmT) was added volume/volume as a negative control to compare to bladder cells exposed to Vat supernatant. Cell damage was determined by lactate dehydrogenase (LDH) release using the LDH-Cytotoxicity Assay Kit II (Biovision, #K313) according to the manufacturer's instructions; the absorbance was measured at an optical density of 495 nm using a microplate reader (Fanizza et al., 2009). The background control (RPMI 1640- medium only) and the lysis control (treatment with Triton X-100) (Sigma, #T8787) were used (Peidaee et al., 2013). All the samples were tested by triplicate (*n* = 3). The results were obtained and analyzed statistically using Dunnett's multiple comparisons tests.

### Evaluation of Vacuole Formation in Bladder Cells Following Exposure to Vat

The concentrated supernatant of the toxin was tested to determine the effect on bladder cells *in vitro* as reported previously for the effect of Vat on chicken fibroblast cell culture (Parreira and Gyles, 2003). The 5637-bladder cell line was grown in 8-well Lab-Tek chambers (Nunc, #Z734853) until ~60% confluence. The monolayers were exposed to 50 μg/ml of the Vat toxin per well with a total volume of 300 μl RPMI 1640 and incubated for 0.5, 1, 3, 6, and 12 h at 37°C with 5% CO_2_ (Greune et al., 2009; Habouria et al., 2019). After incubation, the cells were washed three times with PBS and stained with Giemsa dye (Hu et al., 2015). Vacuole formation was observed by optical microscope (Zeiss, Primo star). A random semiquantitative analysis of the images of the vacuolated cells per well was done, and the results were statistically analyzed using a one-way ANOVA multiple comparisons test.

### Evaluation of Changes on Epithelial Bladder Cell Junctions Induced by Vat

Cultures of bladder cells were grown in 8-well Lab-Tek II chamber slides until reaching 100% confluence. The cultures were exposed to 50 μg/ml of Vat for 6 h, 12 h and 24 h; supernatant from a clone containing the empty vector was used as a negative control. The integrity of the urothelial monolayer was observed by Scanning Electron Microscopy. After exposure to Vat for different time points, samples were fixed with 2.5% Glutaraldehyde for 24 h, after that, the samples were dehydrated using increasing concentrations of ethanol (60, 70, 80, 90, 96, and 100%), incubating for 10 min in each step. At the end, the samples were dried at 37°C/5% CO_2_ for 12 h (Nordestgaard and Rostgaard, 1985). The slide was covered with 100 Å of gold, using Denton Vacuum Desk II. Images were obtained and analyzed under JEOL JSM 5900LV, Scanning Electron Microscope.

The cell-cell distribution of ZO-1 and Occludin, tight junction molecules, were analyzed by immunofluorescence, using the same toxin exposition protocol as described above. Epithelial cells were fixed with 3.7% formaldehyde. After washing with PBS, the cells were permeabilized with 0.1% Triton X-100 for 5 min and then incubated with blocking solution, PBS / 5% BSA for 30 min. To label ZO-1 and Occludin, it was used a primary antibody Anti-ZO-1 (Abcam, #ab221547) and Anti-Occludin (Abcam, #ab216327) respectively, incubating for 2 h at 37°C. Secondary antibody anti-rabbit conjugated with Alexa fluor 488 for ZO-1 (Sigma-Aldrich, #SAB4600387) and Alexa fluor 594 (ThermoFisher, #A-11012) for Occludin. The samples were incubated for 2 h at 37°C. Then, samples were mounted with ProLong Gold (Invitrogen, # P36930) antifade reagent (Bercier et al., 2018). Images were obtained using a confocal microscope (Zeiss, LSM700) with Zen Black software (2012).

### Fluorescence-Based Permeability Assay

Permeability changes following exposure of the bladder cell monolayer with Vat, were evaluated by a paracellular permeability assay using FITC-Dextran 4 (FD4) (Sigma-Aldrich, #60842-46-8) according to the method of Bercier et al. (2018). Briefly, 5637 Bladder cells were seeded and incubated until 100% confluence on Transwell polyester membrane cell culture inserts (Sigma-Aldrich, #CLS3472). The Basolateral space was filled with 300 μl PBS at the start the experiment. The apical chamber was filled with 300 μl of RPMI containing 50 μl/ml of Vat and 1 mg/ml of FD-4. The control was established using the empty vector supernatant in the same amount of cell media and FD4. The permeability measurement was made at 6, 12, and 24 h using a FP-8000 Series Fluorometer. The numeric results were interpreted as Relative Fluorescent Units (Bercier et al., 2018). All the experiments were performed by triplicate and the data analyzed statistically using two-way ANOVA comparisons tests.

### Evaluation of Cell Damage and Cytoskeletal Changes Caused by Exposure to Vat

The F-actin, α-tubulin and Arp3 protein distribution changes produced following Vat treatment were evaluated using bladder cell cultures. Cell monolayers were cultured in 8-well Lab-Tek chambers until 60% confluency was reached and cells were then incubated with 50 μg of the Vat protein for 6 h; as a control to rule out a possible LPS effect on samples, cells were incubated with the toxin simultaneously with 50 μg/ml of polymyxin B (InvivoGen, #1405-20-5) (Tsuzuki et al., 2001; Lu et al., 2017). Cells incubated with 50 μg/ml of heat-inactivated Vat at 95°C for 20 min provided another control (Salvadori et al., 2001; Simon et al., 2018). After incubation, the cells were washed three times with PBS and were fixed.

F-Actin labeling was done by permeabilizing the samples and incubating with Alexa Fluor 488 phalloidin (Invitrogen, #A12379) at 37°C, during 60 min (Guerrero-Barrera et al., 1996). To observe the effect on α-tubulin after Vat incubation, samples were permeabilized and blocked as described above. To label tubulin in cells, anti-α-Tubulin (Sigma, #T5168) at 10 μg/ml was used as the primary antibody and incubated for 2 h at 37°C. This was followed by incubation with a secondary antibody conjugated with Alexa fluor 488 for 2 h at 37°C. The samples were mounted (Invitrogen, # P36930) and images were obtained by confocal microscope.

### Labeling of Acidic Organelles in Bladder Cells After Exposure to Vat

Samples were processed for fluorescence detection of acidic organelles with Lysotracker Deep Red staining (Thermo Fisher, # L12492) (Chen et al., 2015; Magryś et al., 2018). Bladder cells plated on 8-well glass slides were treated with 50 μg/ml of Vat for 6 h. After incubation, cells were washed three time with PBS and were exposed to 300 μl of RPMI added with Lysotracker Deep Red reagent at 10 nM. After 1 h of incubation at 37°C, samples were washed and mounted with Prolong Gold prior to analysis by confocal microscopy (Nagahama et al., 2011).

### *Ex vivo* Culture of Murine Urinary Bladder Exposed to Vat

The animal protocol for this study was approved by the animal ethics committee of the Autonomous University of Aguascalientes, México in accordance with the NIH ethical program. Eight BALB/c female mice were sedated and euthanized to obtain by midline laparotomy urinary bladders. The procedure was developed under sterile conditions (Durnin et al., 2018; Gabella, 2019). In 12-well plates (Corning, #3513) the bladders were placed in RPMI 1640 supplemented with 100 μg/mL streptomycin, 100 U/mL penicillin, 50 mg/L gentamicin. The tissues were exposed to 25, 50, and 100 μg of Vat toxin and incubated for 24 or 48 h at 37°C in 5% CO_2_. The control tissue was incubated for 48 h with supernatant from a clone containing the empty vector. Another control used was simply RPMI 1640 medium for 48 h in order to observe possible changes in the tissue during the cell culture time period (Kannan and Baseman, 2006). Formalin-fixed bladders were dehydrated and cleared automatically with a Histokinette (Leica, #TP1020). Next, the tissues were embedded in paraffin and 5 μm thick sections were obtained (Kim et al., 2010; Najafzadeh et al., 2011). Tissues were stained with hematoxylin and eosin (Prophet et al., 1995) and observed under an optical microscope with a 40X objective (Zeiss, Primo star).

### Statistical Methods

GraphPad Prism 8.0 was the software used to evaluate the quantitative data in this study. Dunnett's multiple comparisons tests were used to analyze the presence of statistically significant differences (*P* ≤ 0.05) between the cells exposed to empty vector supernatant or following exposure to different concentrations of Vat for the LDH release assay. One-way ANOVA, and the multiple comparisons test was used to evaluate the quantity of vacuolated cells per field generated depending on the exposure time to Vat. Two-way ANOVA was used for statistical comparisons of samples from the fluorescence-based cell permeability assay.

## Results

### Cytotoxic Effect of Vat Toxin Measured by Lactate Dehydrogenase Release Assay

**Figure 2:**
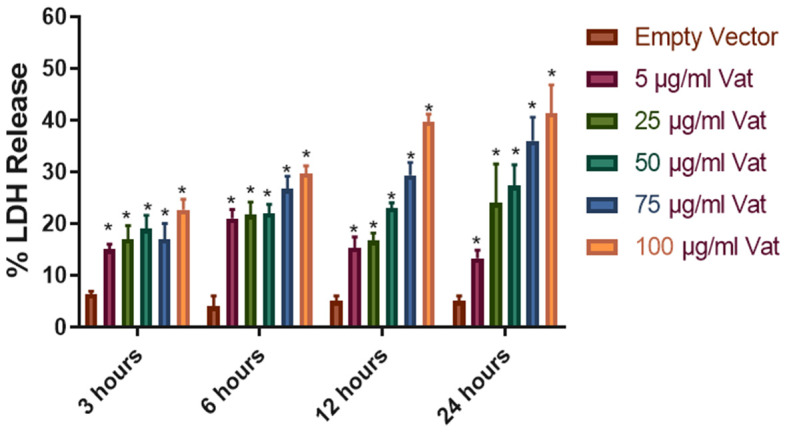
Cellular damage provoked by Vat toxin measured by LDH release. Cells from human urinary bladder cell line 5,637 were exposed during 3, 6, 12, and 24 h to 5, 25, 50, 75, and 100 μg/ml of Vat toxin. As a control supernatant from the empty vector was used. After incubation, LDH in the cell culture supernatant was measured. All toxin concentrations showed statistically significant differences compared to the control in a dose- and time-dependent manner. The percentage of cell damage after toxin exposure resulted in 15 to 47% LDH release (*p ≤ 0.05, two-way ANOVA, Dunnett's test).

LDH release from cells to the extracellular media is considered an indicator of cell membrane integrity damage (Fanizza et al., 2009; Radin et al., 2011). Vat treatment of bladder cells with different toxin concentrations (5, 25, 50, 75, 100 μg/ml) at different times (3, 6, 12, and 24 h) showed statistically significant differences in comparison with the *E. coli* empty vector supernatant treatment (*P* ≤ *0.05*) (Figure 2). LDH released to the media was dependent on the time of exposure and concentration of Vat.

### Vacuole Formation in Bladder Cells Exposed to Vat

**Figure 3:**
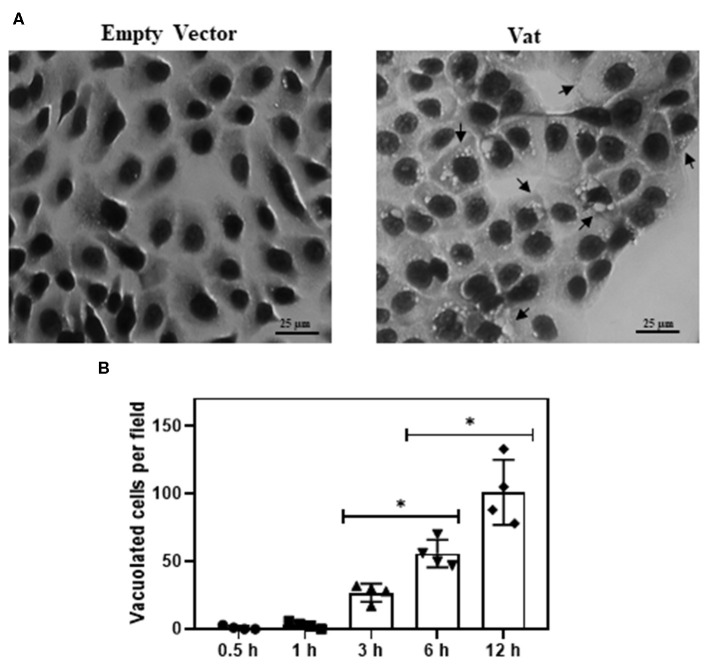
Effect of Vat in human urinary bladder cells 5637 at different time of exposition **(A)** Control cells exposed to empty vector for 6 h, showing normal cell shape and distribution on the monolayer. The cells incubated for 6 h with 50 μg/ml of Vat toxin, exhibit cytoplasmic vacuole formation (black arrows). **(B)** Plotted results of the vacuole formation showed a significative difference between 3 to 6 h and 6 to 12 h. (*p ≤ 0.05, ANOVA, Dunnett's).

The bladder cells exposed to Vat during 6 h showed vacuole formation that increased in numbers with the time of exposition (Figure 3A). There were no vacuoles observed following exposure to toxin for 0.5 or 1 h. Statistically significant vacuole production (Supplementary Image 1) was found after 3 h of exposure to the toxin (Figure 3B).

### Evaluation of Changes to Epithelial Cell Junctions Following Exposure to Vat

**Figure 4:**
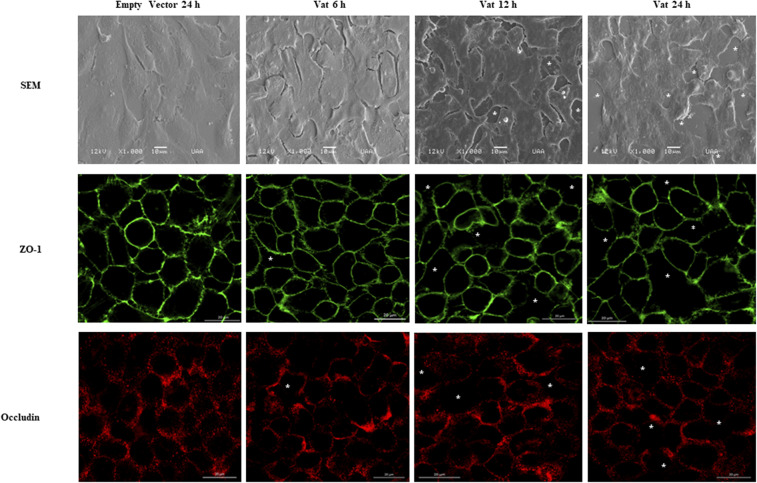
Modification of cell junctions of the bladder urothelial monolayer after incubation with Vat. Analysis by scanning electron microscopy revealed loss of integrity of the cell monolayer (White asterisks) starting at 6 h. The changes in the monolayer are more visible at 12 and 24 h after exposure to the toxin in comparison to the control. The immunofluorescence labeling of ZO-1 and Occludin proteins show cell junction disruption in the cell monolayer.

Scanning Electronic Microscopy analysis of the bladder cell monolayer, showed increased spacing between cells when it was exposed to Vat, as well as changes in cell morphology (Figure 4). After 6 h of treatment, these spaces appeared in the monolayer when compared to the control cells. This damage increased with time of exposure and resembled alterations between cell-cell junctions. In order to characterize cellular alterations due to Vat, the localization of two important tight junction molecules, ZO-1 and Occludin, were evaluated. Vat induced discontinuities in the pattern of distribution of both ZO-1 and Occludin in bladder epithelial cells following 12 and 24 h of treatment (Figure 4).

### Fluorescence-Based Permeability Assay for Epithelial Bladder Monolayer Cell Culture Treated With Vat Toxin

**Figure 5:**
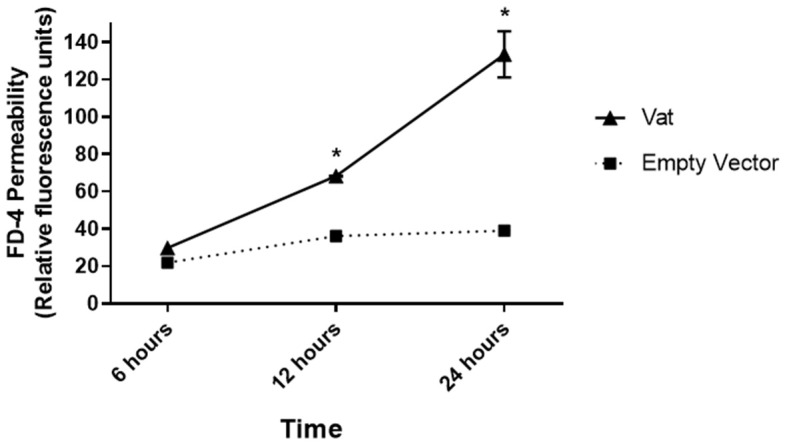
Fluorescence-based permeability assay of a bladder epithelial cell monolayer treated with Vat toxin. The quantitative method showed changes in the permeability of the monolayer exposed to the toxin. Comparison between the Vat toxin and the empty vector control supernatant had statistically significant differences at 6 and 12 h of incubation (*p ≤ 0.05, two-way ANOVA).

Changes in monolayer integrity were confirmed by the permeability assay. The quantification of FD4 fluorescence in the supernatants of basolateral transwell chambers showed a significative difference in permeability at 12 and 24 h of toxin exposure with respect to the control (Figure 5). The fluorescence increased by ~2-fold from 12 to 24 h of exposure.

### Evaluation of Cell Damage Induced by Vat Through Cytoskeletal Changes

**Figure 6:**
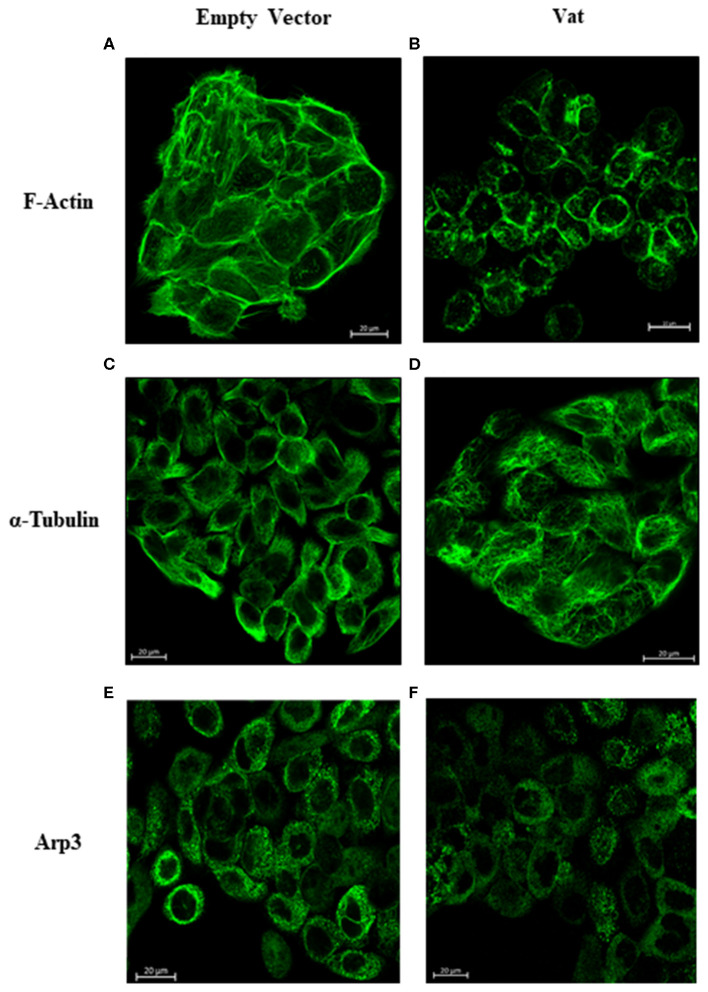
Changes in the distribution of F-actin, α-Tubulin, and Arp3 cytoskeletal proteins detected after Vat treatment of bladder cells observed by confocal microscopy. The negative control cells **(A)** showed a normal distribution of actin stress fibers with a normal cell shape and appearance. After exposure to Vat **(B)** the cells acquired a rounded shape with loss of the cytoplasmic actin stress fibers. Normal tubulin distribution was observed in the control cells **(C)** with its presence throughout the cell. Once cells were exposed to Vat **(D)** the distribution of tubulin was altered. Arp3 demonstrated a cytoplasmic dotted distribution **(E)** on the untreated cells, and there was no evident change in Arp3 distribution when cells were treated with Vat **(F)**.

Bladder cell cultures exposed to Vat were analyzed for alterations of the cytoskeleton components actin and tubulin by confocal microscopy. After 6 h of exposure to Vat toxin, cells labeled with phalloidin, revealed changes in F-actin distribution with a diffuse pattern (Figure 6B) resulting in round cell shape, loss of integrity of stress fibers, and disruption of cell-cell interactions. In the control cells treated with the empty vector supernatant, the F-actin cytoskeleton was intact with well-defined stress fibers distributed along with the cytoplasm (Figure 6A). In control cells, tubulin showed mainly a peripheral distribution and a normal cell shape (Figure 6C). When cells were treated with Vat toxin, this pattern changed, and tubulin was more diffuse at the cell surface, and correlated with the altered morphology of the cells (Figure 6D). Toxin activity was validated using control samples, testing the cells with heat-inactivated toxin and by the addition of polymyxin B to inhibit any potential cellular changes that might be caused by traces of LPS present in the culture supernatants (Supplementary Image 2).

### Labeling of Acidic Organelles After Exposure to Vat

**Figure 7:**
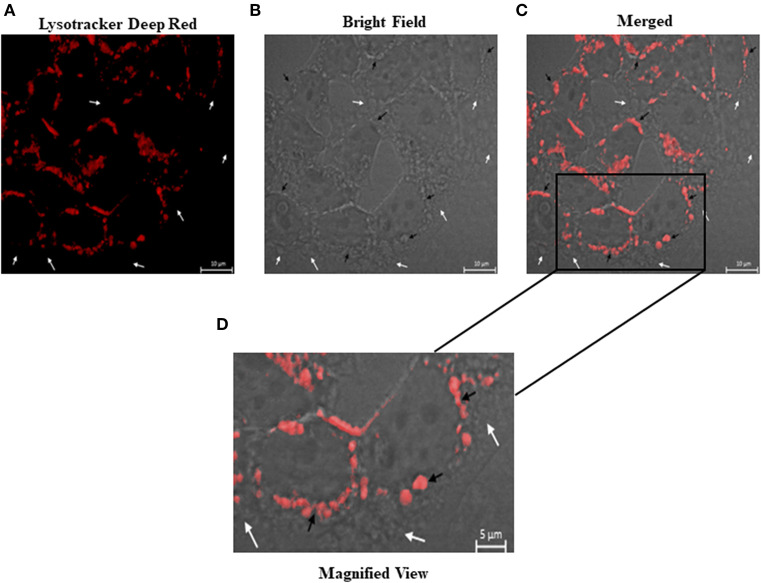
Characterization of the vacuoles produced in bladder epithelial cells after treatment with Vat. After 12 h of Vat exposure, samples were labeled with Lysotracker deep red to identify acidic organelles **(A–C)**. The vacuoles that incorporated Lysotracker reagent (Black arrows) show a red color. Some other vacuoles were identified in the samples which did not have an acidic content (White arrows) **(C,D)**.

Acidic organelles were found using Lysotracker Deep Red. Figure 7 shows cells after 12 h of Vat treatment (Figure 7B), with the distribution of acidic organelles. A merged image combining bright field and fluorescence (Figure 7C) identifies cells containing multiple vacuoles that are not fluorescent (white arrows), whereas acidic organelles are shown with black arrows. Supplementary Image 3 shows that acidic organelles are also produced by control cells. By contrast the vacuoles produced by Vat are not labeled with Lysotracker.

### *Ex vivo* Culture of Murine Urinary Bladder Tissue Exposed to Vat

**Figure 8:**
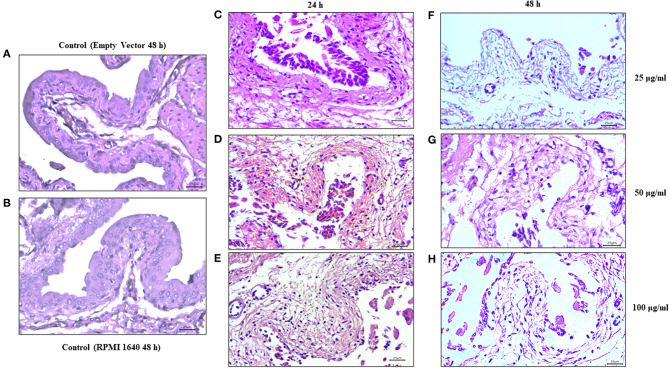
Effect of Vat on urinary bladder tissues. Mouse urinary bladders were incubated *ex vivo* with 25, 50, and 100 μg/ml of Vat toxin supernatant for 24 h or for 48 h. After 24 h of Vat treatment **(C–E)**, detachment of the urothelium from the underlying connective tissue was observed (Black arrows). This effect was dependent on the toxin concentration. After 48 h of incubation, alterations in the lamina propria (Black asterisk) were visible on bladder samples with a loose and disorganized connective tissue **(F–H)**. Those changes were compared with bladders exposed to the empty vector supernatant and the control containing only RPMI 1640 medium **(A,B)**.

Bladder tissues exposed to different concentrations of Vat for 24 and 48 h showed partial cell desquamation of the urothelial cells (Black arrow) in comparison with the controls. This effect was dependent on toxin concentration (Figure 8). With 25 μg/ml of toxin at 24 h (Figure 7C), the most superficial urothelial cells showed exfoliation from the underlying tissue. Treatment with 50 μg/ml of Vat for 24 h (Figure 8D) caused extended changes in epithelial cell integrity, affecting deeper stratum. Higher concentrations of Vat resulted in a thinner urothelium in comparison with other samples, this suggests that Vat caused a loss of normal integrity of the epithelial cell barrier. Also, Vat treatment showed an alteration of the urinary bladder lamina propria. After 48 h of incubation with 25 μg/ml of Vat, the transitional epithelium was absent and the lamina propria was loose and disorganized (Figure 8F). Treatment with 50 μg/ml of toxin increased damage to the lamina propria, resulting in loss of the normal bladder structure (Figure 8G). Similar results were observed with 100 μg/ml of Vat at 48 h (Figure 8H). Tissue changes were compared with bladder exposed to supernatant from bacteria containing only the empty vector or only with RPMI 1640 cell culture medium for 48 h in order to validate the *ex vivo* experiments and the action of Vat toxin on tissues (Figures 8A,B).

### Discussion

Exposure of bladder cells to the Vacuolating autotransporter toxin caused vacuole formation, and these changes resembled cytological changes observed previously in avian cell models (Parreira and Gyles, 2003; Nichols et al., 2016). Also, cell rounding of the bladder cells and the alteration in cell-cell contact could result in the loss of integrity of the urothelial barrier. This epithelial disruption has also been observed with another vacuolating toxin, VacA, from *Helicobacter pylori* (Tombola et al., 1999; de Bernard et al., 2004; Bercier et al., 2018).

Lactate Dehydrogenase (LDH) is a cytoplasmic enzyme that is released into the extracellular medium when cell membrane integrity is compromised. Time of exposure and dose effect on cytotoxic activity of Vat was assessed by LDH release and suggests that a high concentration and exposure time of at least 6 h was required to achieve a high level (>50%) of cytotoxicity for bladder cells *in vitro*. These results in comparison to other bacterial toxins from *Escherichia coli, Helicobacter pylori* and *Clostridioides difficile* toxin (Grossmann et al., 2000; Roberts et al., 2001; Radin et al., 2011), suggest that Vat likely plays a subtle role in the pathogenesis of UTIs (Roberts et al., 2001; Peterson et al., 2009).

Cell damage can also be evaluated through cytoskeletal changes such as distribution of F-actin and tubulin that have an effect on cell morphology and cell-cell association. Alfaro-Aco and Petry (2015) have proposed that actin and tubulin cytoskeleton components play a critical role in cytoprotection, intercellular junction maintenance, shape definition and intracellular vesicular transport (Tang et al., 2014; Gefen and Weihs, 2015; Tran and Ten Hagen, 2017). Damage to these cytoskeletal components, as observed after the exposure to Vat, affects the normal cell distribution and morphology *in vitro*. The alteration and redistribution of F-actin and α-tubulin in the cytoskeleton correlated with the morphological changes in cells, a decrease in monolayer integrity and the desquamation of cells from the substrate, a phenomenon similar to what occurs following exposure of cells to other SPATEs (Dautin, 2010; Liévin-Le Moal et al., 2011; Glotfelty et al., 2014; Gasic and Mitchison, 2019).

Vacuole formation within mammalian cells affects cell homeostasis. A variety of secreted bacterial toxins can induce vacuole production in cells with different functions in its pathogenesis in the host (Lee et al., 2015; Shubin et al., 2016; Magryś et al., 2018). In order to determine the nature of the vacuoles generated by the Vat toxin, the presence of acidic organelles was determined in bladder cells. The results obtained in this study by the overlaying of fluorescence confocal microscopy and bright field image, reveals acidic content in some vacuoles generated by Vat as well as others vacuoles with non-acidic characteristics inside of them (Nagahama et al., 2011; Chen et al., 2015). The non-lysosomal nature of some vacuoles in bladder cells treated with Vat is of interest and merits further investigation (Appelqvist et al., 2013; Ram and Ramakrishna, 2014; Shubin et al., 2016).

Finally, our *ex vivo* experiment suggests that Vat can induce detachment of epithelial cells comprising the urothelium of the bladder, possibly as a direct effect of the toxin on the adhesion molecules of the cell surface or due to an indirect effect caused by changes in the morphology of the cells as a consequence of alterations to the cytoskeleton. Further, the lamina propia underlying the urothelium of the bladder was affected by Vat at the highest concentration. This suggests that some connective tissue molecules may be targeted by the toxin. Proteolytic activity of Vat has been shown to exhibit an elastase-like activity (Habouria et al., 2019). This result is in agreement with our *in vitro* experiments in which we observed the loss of intercellular contacts of cells from the monolayer and in its integrity showed with the permeability assay.

In conclusion, the effect of the vacuolating autotransporter toxin from *Escherichia coli* was investigated by using a bladder epithelial cell model. Cytotoxicity of Vat was dose-dependent and suggested that Vat most likely acts as a cytopathic toxin that alters bladder cell function with a limited degree of cell death. Vat caused vacuole formation on bladder cells similar to the cytopathic effects that were previously reported following exposure of avian cells to *E. coli* culture supernatants containing Vat. Also, the Vat toxin caused alterations on cell junctions, affecting monolayer integrity, causing redistribution of tight junction proteins and increasing urothelial cell permeability. Furthermore, redistribution of actin and tubulin in bladder epithelial cells occurred simultaneously with morphological changes on cells. In addition, our results suggest that some vacuoles on epithelial cells induced by Vat have not acidic content. Finally, our *ex vivo* experiments on murine bladder demonstrated that Vat caused alterations of the urothelium and lamina propria of the bladder. It will be of future interest to investigate whether Vat targets an adhesion molecule on the epithelial cell surface or a specific component of the lamina propria. As well, characterization of the composition of the vacuoles induced by Vat may provide further evidence of how this toxin contributes to the pathogenesis of UTIs caused by UPEC.

## Data Availability Statement

All datasets generated for this study are included in the article/Supplementary Material.

## Ethics Statement

This study was reviewed and approved by the ethics committee for the use of animals in the teaching and research of The Autonomous University of Aguascalientes.

## Author Contributions

JD was the primary author of the manuscript. CD and PP contributed to optimization of protein production and revision of the manuscript. FA-G provided expertise in statistical analysis. EH-C advised for the planning and design of some experiments. AS contributed to the development of experimental work. AG-B proposed the line of research and was responsible for major funding of the project.

## Conflict of Interest

The authors declare that the research was conducted in the absence of any commercial or financial relationships that could be construed as a potential conflict of interest.

## Supplementary Material

Supplementary Image 1 Kinetics of vacuole formation induced by Vat toxin on human urinary bladder cell line 5,637. Kinetics of vacuole formation (Black arrows) showed cytoplasmic vacuoles after 3 h of toxin exposure, with vacuole formation increasing over time.

Supplementary Image 2 Distribution of F-actin, α-Tubulin, and Arp3 cytoskeletal proteins detected after treatment of bladder epithelial cells with Vat observed by confocal microscopy. The negative control cells **(A)** show a normal distribution of actin stress fibers with a homogeneous pattern. A similar pattern was observed in samples exposed to 50 μg of heat-inactivated Vat toxin **(B)**. After exposure to Vat **(C)**, the cells acquired a rounded shape with a loss of the cytoplasmic actin stress fibers. This effect was also observed in cells treated with Vat together with polymyxin B **(D)**. Tubulin was distributed throughout the cell in control cells **(E)** with its presence throughout the cell. Cells incubated with the toxin after heat inactivation have a similar tubulin pattern as those treated with the empty vector supernatant **(F)**. Once cells were exposed to Vat **(G)**, the tubulin pattern showed cytoplasmic rearrangement resembling the morphological changes in cell shape **(H)**. The presence of Polymyxin B in the cell culture did not alter the effect of the toxin on cells. Arp3 had a cytoplasmic dotted distribution **(I)** in untreated control cells. This was also the case with cells exposed to the inactivated toxin **(J)**. Cells after treatment with Vat **(K)** with or without polymyxin B **(L)**, showed a homogeneous cytoplasmic distribution of Arp3 in contrast to the control cells.

Supplementary Image 3 Characterization of the vacuoles in bladder epithelial cells treated with Vat. After exposure to Vat toxin, cells were stained with Lysotracker deep red and visualized. Vacuoles with acidic content (Black arrows) with a perinuclear distribution were observed and other vacuoles without lysotracker staining were also observed (White arrows). The samples exposed to supernatant from bacteria contain the empty vector did not produce vacuoles (Bright-field microscopy), and the slight lysotracker staining may indicate a basal level of lysosomes in these cells.
