# Supplementary figures and images for: The Vacuolating Autotransporter Toxin (Vat) of Escherichia coli Causes Cell Cytoskeleton Changes and Produces Non-lysosomal Vacuole Formation in Bladder Epithelial Cells
Source: Front Cell Infect Microbiol. 2020 Jun 26;10:299. doi: 10.3389/fcimb.2020.00299 (PMC7332727; doi:10.3389/fcimb.2020.00299)

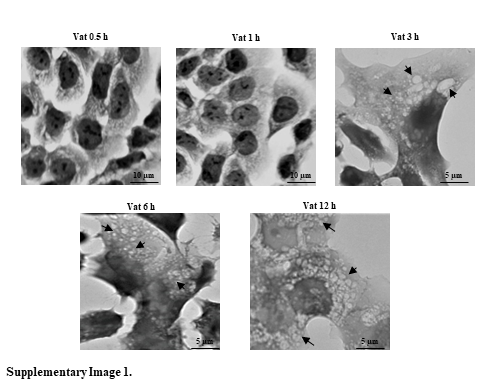

Supplement: Supplementary Image 1 — Kinetics of vacuole formation induced by Vat toxin on human urinary bladder cell line 5,637. Kinetics of vacuole formation (Black arrows) showed cytoplasmic vacuoles after 3 h of toxin exposure, with vacuole formation increasing over time. [file Data_Sheet_1.zip › Figure S1.TIF]

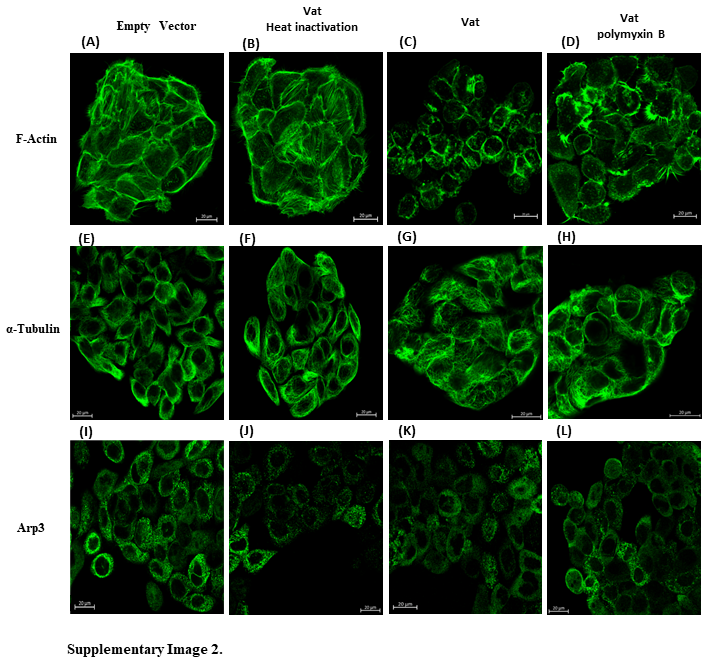

Supplement: Supplementary Image 1 — Kinetics of vacuole formation induced by Vat toxin on human urinary bladder cell line 5,637. Kinetics of vacuole formation (Black arrows) showed cytoplasmic vacuoles after 3 h of toxin exposure, with vacuole formation increasing over time. [file Data_Sheet_1.zip › Figure S2.TIF]

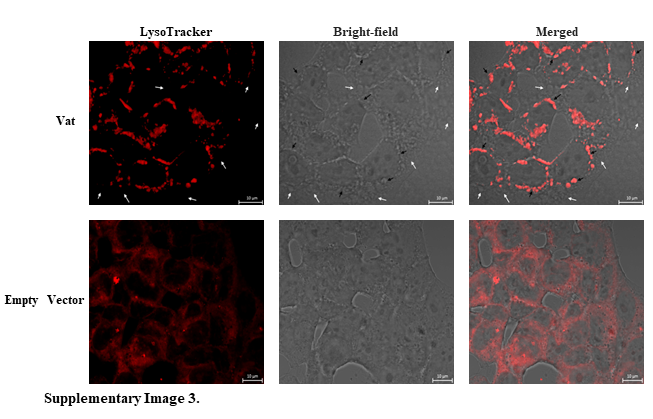

Supplement: Supplementary Image 1 — Kinetics of vacuole formation induced by Vat toxin on human urinary bladder cell line 5,637. Kinetics of vacuole formation (Black arrows) showed cytoplasmic vacuoles after 3 h of toxin exposure, with vacuole formation increasing over time. [file Data_Sheet_1.zip › Figure S3.TIF]
